# The genome sequence of the long-spined sea scorpion, *Taurulus bubalis *(Euphrasén, 1786)

**DOI:** 10.12688/wellcomeopenres.17356.1

**Published:** 2021-11-08

**Authors:** Sophie Potter, Darwin Tree of Life Barcoding collective, Wellcome Sanger Institute Tree of Life programme, Wellcome Sanger Institute Scientific Operations: DNA Pipelines collective, Tree of Life Core Informatics collective, Darwin Tree of Life Consortium

**Affiliations:** 1 Tree of Life, Wellcome Sanger Institute, Cambridge, UK

**Keywords:** Taurulus bubalis, long-spined sea scorpion, genome sequence, chromosomal

## Abstract

We present a genome assembly from an individual female *Taurulus bubalis *(the long-spined sea scorpion; Chordata; Actinopteri; Perciformes; Cottidae). The genome sequence is 615 megabases in span. The complete assembly is scaffolded into 21 chromosomal pseudomolecules.

## Species taxonomy

Eukaryota; Metazoa; Chordata; Craniata; Vertebrata; Euteleostomi; Actinopterygii; Neopterygii; Teleostei; Neoteleostei; Acanthomorphata; Eupercaria; Perciformes; Cottioidei; Cottales; Cottidae; Taurulus; *Taurulus bubalis* (Euphrasén, 1786) (NCBI:txid61643).

## Background

The long-spined sea scorpion ( *Taurulus bubalis*, Perciformes: Cottidae), also known as the longspined bullhead or fatherlasher, is named for the distinctive long spine found on its cheek (above the pectoral fin and behind the eye). It is a rocky shore fish found throughout western European waters and around all coasts of Britain and Ireland, from the shore to around 30 m depth. They are also occasionally seen in the Mediterranean. Adult fish can reach around 20 cm long and have a broad head with a large mouth. They are sometimes confused with the bull rout, *Myoxocephalus scorpius* (also known as the short-spined scorpion fish), but adult bull rout are much larger and lack the distinctive cheek spine ( Neal, 2008). Long-spined sea scorpions are ambush predators with cryptic coloration and have a varied diet, primarily focused on crustaceans, but molluscs, fish and polychaetes are also consumed. Most prey are swallowed whole. Individuals grow rapidly during the first two years of life, and may begin to spawn during their second year. All individuals will have spawned by their third year, with spawning occurring between December and March ( King & Fives, 1983).

The long-spined sea scorpion has a notable behavioural response to changing environmental conditions: when oxygen tension is lower, individuals will emerge from the water and climb onto the land where they breathe air. They are not, however, very mobile once they have emerged ( Davenport & Woolmington, 1981). The long-spined sea scorpion has been classified as “Least Concern” on the IUCN red list ( Lorance *et al.*, 2014).

## Genome sequence report

**Figure 1.**
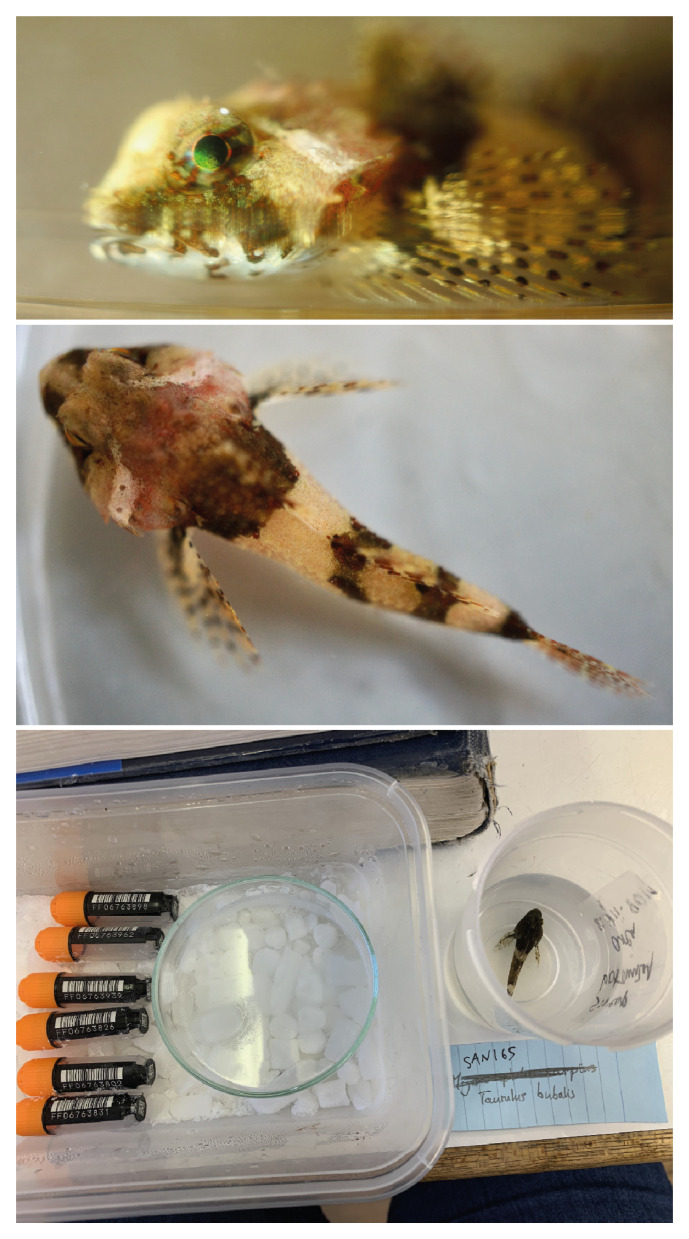
Images of the fTauBub2 specimen taken prior to preservation and processing. The bottom image includes the FluidX storage tubes, length 43.9 mm, for scale.

The genome was sequenced from one *T. bubalis* of unknown sex collected from Farland Point, Great Cumbrae, North Ayrshire, UK (latitude 55.746815, longitude -4.914907) ( Figure 1). A total of 38-fold coverage in Pacific Biosciences single-molecule long reads and 51-fold coverage in 10X Genomics read clouds were generated. Primary assembly contigs were scaffolded with chromosome conformation Hi-C data. Manual assembly curation corrected 161 missing/misjoins and removed 6 haplotypic duplications, reducing the assembly length by 0.28% and the scaffold number by 86.10%, and increasing the scaffold N50 by 31.71%.

**Figure 2.**
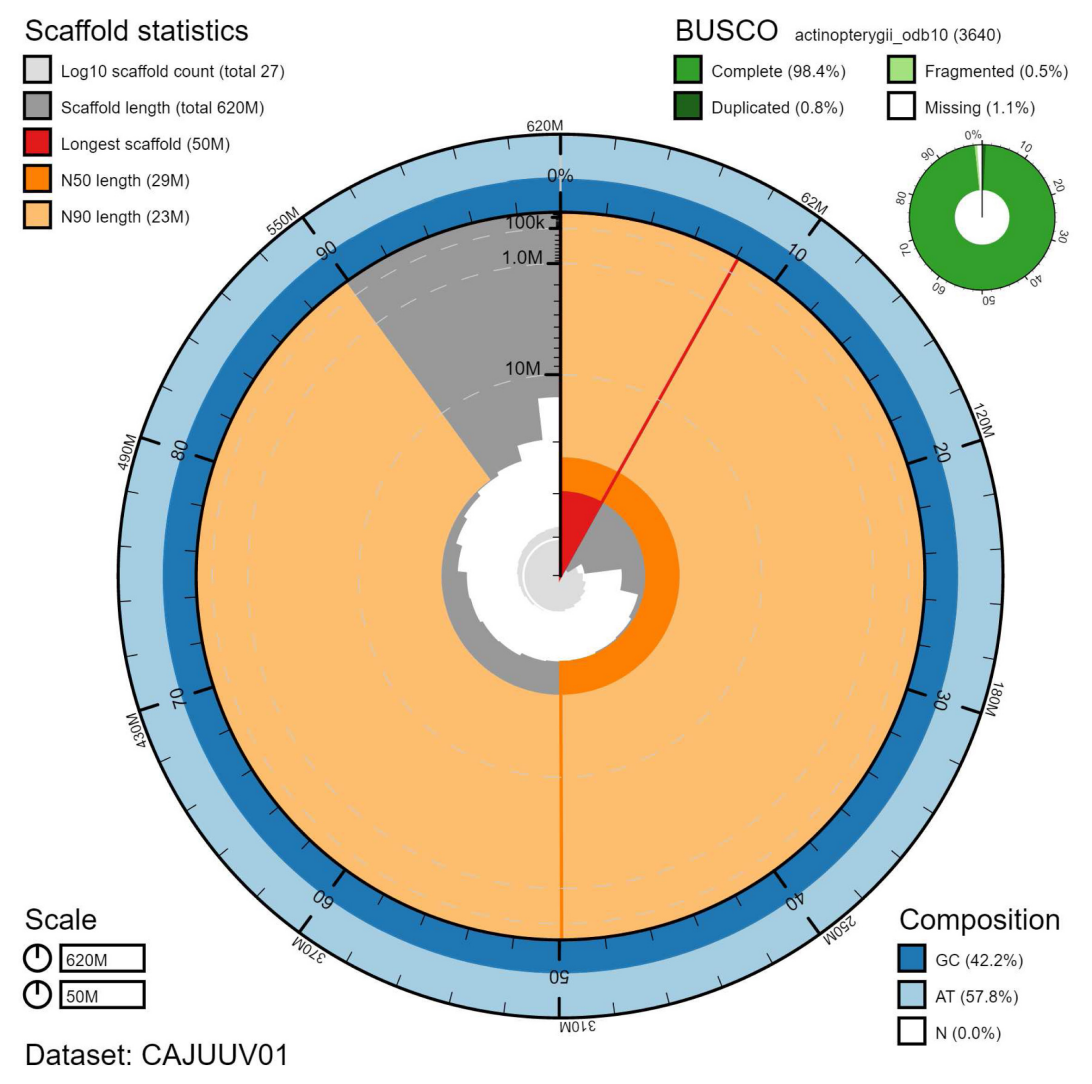
Genome assembly of *Taurulus bubalis*, fTauBub2.1: metrics. The BlobToolKit Snailplot shows N50 metrics and BUSCO gene completeness. The main plot is divided into 1,000 size-ordered bins around the circumference with each bin representing 0.1% of the 615,153,901 bp assembly. The distribution of chromosome lengths is shown in dark grey with the plot radius scaled to the longest chromosome present in the assembly (50,132,026 bp, shown in red). Orange and pale-orange arcs show the N50 and N90 chromosome lengths (29,138,660 and 22,724,154 bp), respectively. The pale grey spiral shows the cumulative chromosome count on a log scale with white scale lines showing successive orders of magnitude. The blue and pale-blue area around the outside of the plot shows the distribution of GC, AT and N percentages in the same bins as the inner plot. A summary of complete, fragmented, duplicated and missing BUSCO genes in the actinopterygii_odb10 set is shown in the top right. An interactive version of this figure is available at https://blobtoolkit.genomehubs.org/view/fTauBub2.1/dataset/CAJUUV01/snail.

The final assembly has a total length of 615 Mb in 26 sequence scaffolds with a scaffold N50 of 29.1 Mb ( Table 1). The complete assembly sequence was assigned to 21 chromosomal-level scaffolds, representing 21 autosomes (numbered by sequence length) ( Figure 2– Figure 5; Table 2). The assembly has a BUSCO v5.1.2 ( Manni *et al.*, 2021) completeness of 98.4% (single 97.6%, duplicated 0.8%) using the actinopterygii_odb10 reference set. While not fully phased, the assembly deposited is of one haplotype. Contigs corresponding to the second haplotype have also been deposited.

**Table 1.** Genome data for *Taurulus bubalis*, fTauBub2.1.

*Project accession data*	
Assembly identifier	fTauBub2.1
Species	*Taurulus bubalis*
Specimen	fTauBub2
NCBI taxonomy ID	NCBI:txid61643
BioProject	PRJEB45118
BioSample ID	SAMEA7522994
Isolate information	Unknown sex, muscle (genome assembly), fin (RNA-Seq), gill (Hi-C)
*Raw data accessions*	
PacificBiosciences SEQUEL II	ERR6412369
10X Genomics Illumina	ERR6054766-ERR6054769
Hi-C Illumina	ERR6054770
Illumina polyA RNA-Seq	ERR6286720
*Genome assembly*	
Assembly accession	GCA_910589615.1
Accession of alternate haplotype	GCA_910589325.1
Span (Mb)	615
Number of contigs	268
Contig N50 length (Mb)	13.0
Number of scaffolds	26
Scaffold N50 length (Mb)	22.1
Longest scaffold (Mb)	50.1
BUSCO * genome score	C:98.4%[S:97.6%,D:0.8%],F:0.5%, M:1.1%,n:3640

*BUSCO scores based on the actinopterygii_odb10 BUSCO set using v5.1.2. C= complete [S= single copy, D=duplicated], F=fragmented, M=missing, n=number of orthologues in comparison. A full set of BUSCO scores is available at https://blobtoolkit.genomehubs.org/view/fTauBub2.1/dataset/CAJUUV01/busco.

**Figure 3.**
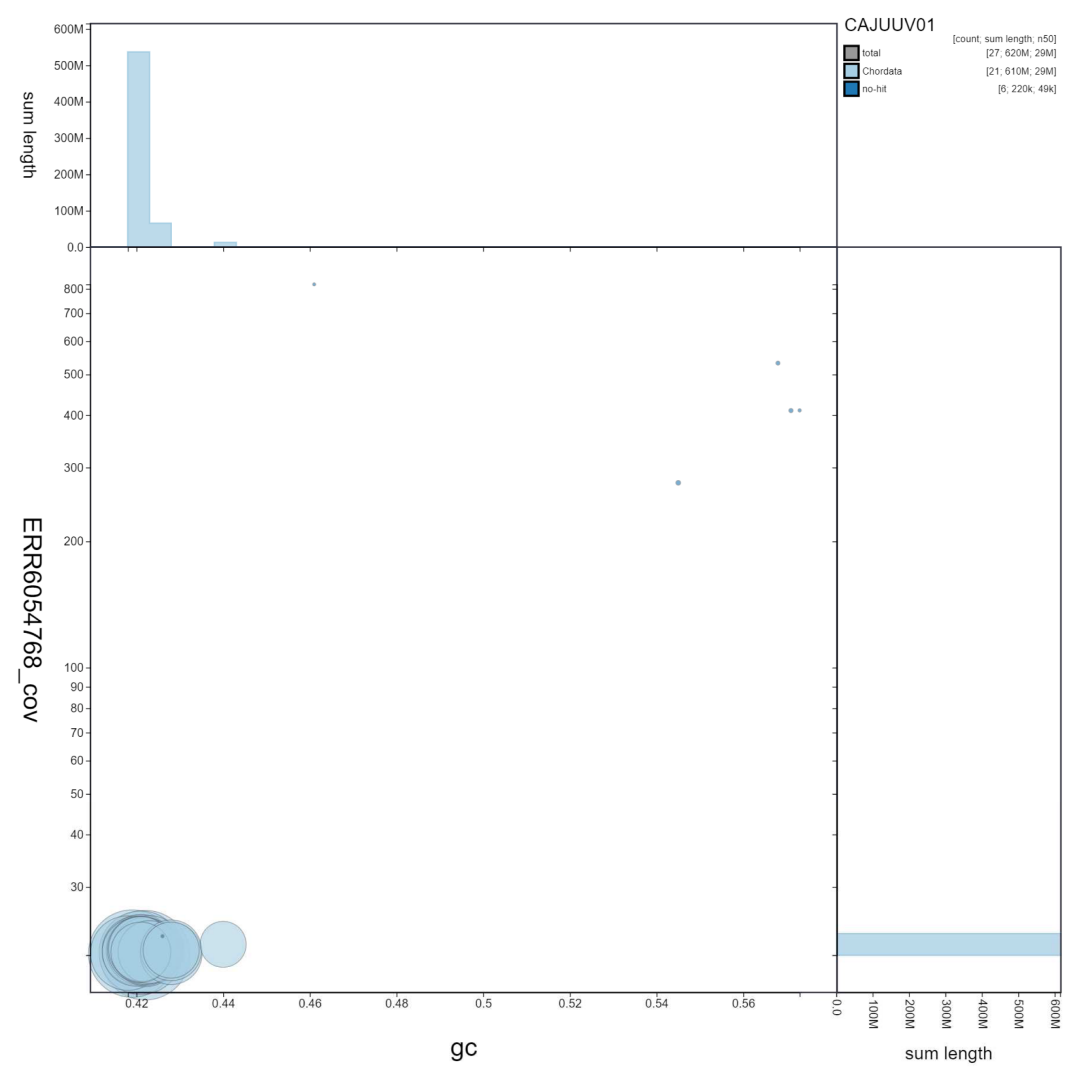
Genome assembly of *Taurulus bubalis*, fTauBub2.1: GC coverage. BlobToolKit GC-coverage plot. Scaffolds are coloured by phylum. Circles are sized in proportion to scaffold length Histograms show the distribution of scaffold length sum along each axis. An interactive version of this figure is available at https://blobtoolkit.genomehubs.org/view/fTauBub2.1/dataset/CAJUUV01/blob.

**Figure 4.**
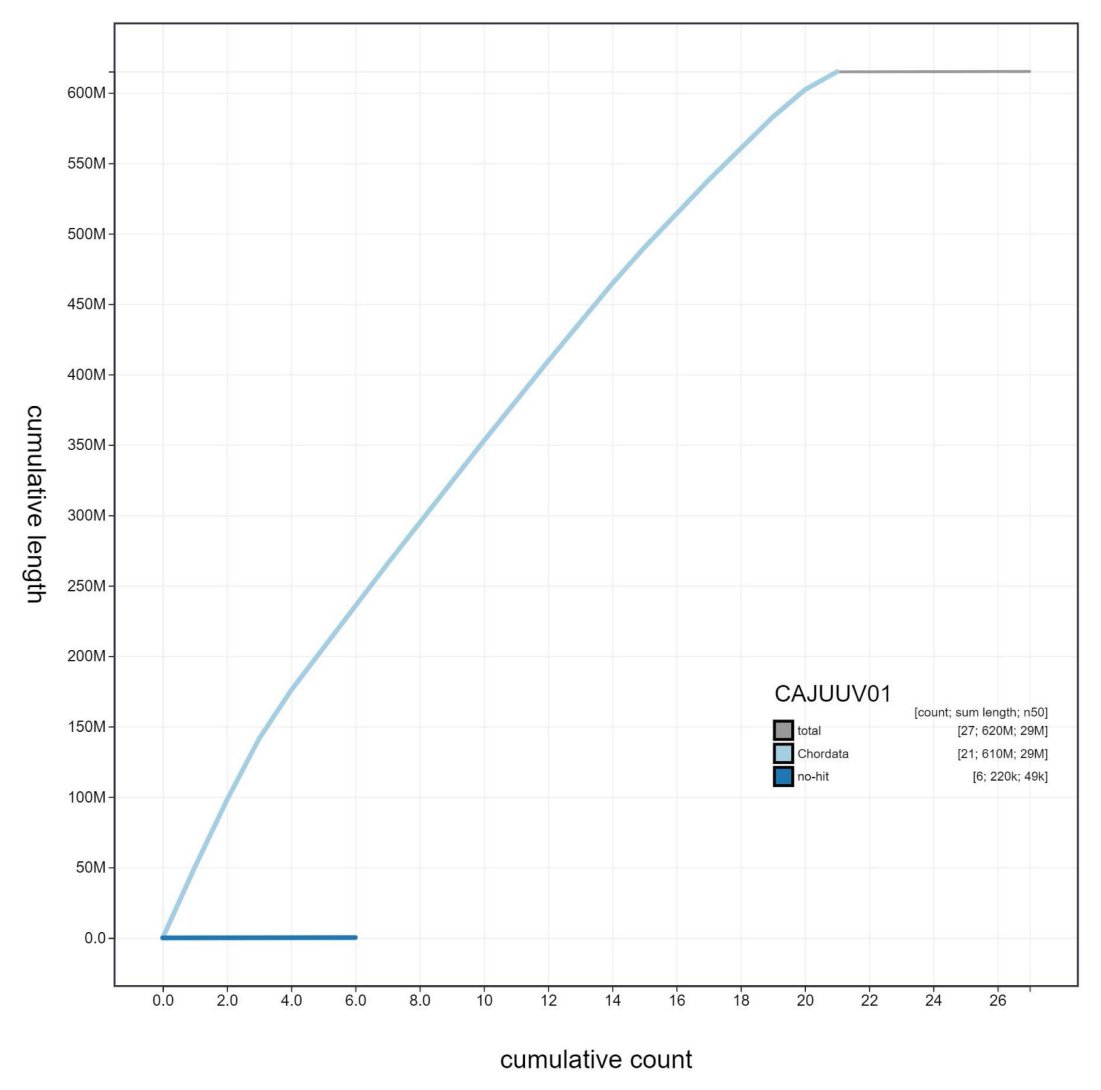
Genome assembly of *Taurulus bubalis*, fTauBub2.1: cumulative sequence. BlobToolKit cumulative sequence plot. The grey line shows cumulative length for all scaffolds. Coloured lines show cumulative lengths of scaffolds assigned to each phylum using the buscogenes taxrule. An interactive version of this figure is available at https://blobtoolkit.genomehubs.org/view/fTauBub2.1/dataset/CAJUUV01/cumulative.

**Figure 5.**
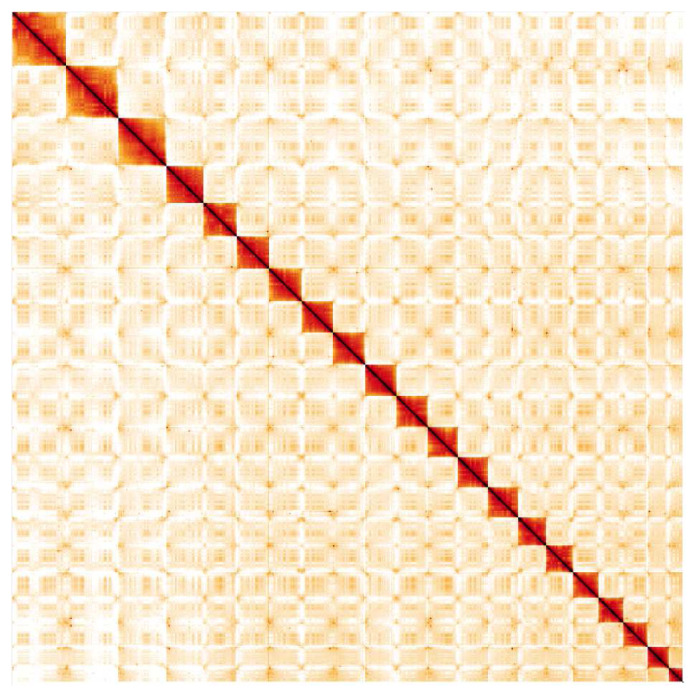
Genome assembly of *Taurulus bubalis*, fTauBub2.1: Hi-C contact map. Hi-C contact map of the fTauBub2.1 assembly, visualised in HiGlass.

**Table 2.** Chromosomal pseudomolecules in the genome assembly of *Taurulus bubalis*, fTauBub2.1.

INSDC accession	Chromosome	Size (Mb)	GC%
OU342711.1	1	50.13	42.2
OU342712.1	2	47.70	41.9
OU342713.1	3	43.50	42.1
OU342714.1	4	34.46	41.8
OU342715.1	5	30.39	42.1
OU342716.1	6	29.76	42.1
OU342717.1	7	29.55	42.2
OU342718.1	8	29.27	42
OU342719.1	9	29.14	42.1
OU342720.1	10	28.94	42
OU342721.1	11	28.35	42.2
OU342722.1	12	28.17	42.3
OU342723.1	13	27.68	42.2
OU342724.1	14	27.45	42.1
OU342725.1	15	25.62	42.1
OU342726.1	16	24.36	42.3
OU342727.1	17	23.58	42.8
OU342728.1	18	22.72	42.8
OU342729.1	19	22.07	42.1
OU342730.1	20	19.29	42.8
OU342731.1	21	12.81	44
OU342732.1	MT	0.02	46.2
-	Unplaced	0.20	54.8

## Methods

### Sample acquisition and nucleic acid extraction

A single *T. bubalis* of unknown sex (fTaubBub2) was collected by hand from Farland Point, Great Cumbrae, North Ayrshire, UK (latitude 55.746815, longitude -4.914907) by Richard Durbin, University of Cambridge/Wellcome Sanger Institute. The sample was identified by the same individual and preserved on dry ice.

DNA was extracted at the Tree of Life laboratory, Wellcome Sanger Institute. The fTauBub2 sample was weighed and dissected on dry ice with tissue set aside for Hi-C and RNA sequencing. Muscle tissue was cryogenically disrupted to a fine powder using a Covaris cryoPREP Automated Dry Pulveriser, receiving multiple impacts. Fragment size analysis of 0.01–0.5 ng of DNA was then performed using an Agilent FemtoPulse. High molecular weight (HMW) DNA was extracted using the Qiagen MagAttract HMW DNA extraction kit. Low molecular weight DNA was removed from a 200-ng aliquot of extracted DNA using 0.8X AMpure XP purification kit prior to 10X Chromium sequencing; a minimum of 50 ng DNA was submitted for 10X sequencing. HMW DNA was sheared into an average fragment size between 12–20 kb in a Megaruptor 3 system with speed setting 30. Sheared DNA was purified by solid-phase reversible immobilisation using AMPure PB beads with a 1.8X ratio of beads to sample to remove the shorter fragments and concentrate the DNA sample. The concentration of the sheared and purified DNA was assessed using a Nanodrop spectrophotometer and Qubit Fluorometer and Qubit dsDNA High Sensitivity Assay kit. Fragment size distribution was evaluated by running the sample on the FemtoPulse system.

RNA was extracted from fin tissue in the Tree of Life Laboratory at the WSI using TRIzol (Invitrogen), according to the manufacturer’s instructions. RNA was then eluted in 50 μl RNAse-free water and its concentration assessed using a Nanodrop spectrophotometer and Qubit Fluorometer using the Qubit RNA Broad-Range (BR) Assay kit. Analysis of the integrity of the RNA was done using Agilent RNA 6000 Pico Kit and Eukaryotic Total RNA assay.

### Sequencing

Pacific Biosciences HiFi circular consensus and 10X Genomics Chromium read cloud sequencing libraries were constructed according to the manufacturers’ instructions. Poly(A) RNA-Seq libraries were constructed using the NEB Ultra II RNA Library Prep kit. Sequencing was performed by the Scientific Operations core at the Wellcome Sanger Institute on Pacific Biosciences SEQUEL II (HiFi), Illumina HiSeq X (10X) and Illumina HiSeq 4000 (RNA-Seq) instruments. Hi-C data were generated from gill tissue using the Arima v2 Hi-C kit and sequenced on HiSeq X.

### Genome assembly

Assembly was carried out with Hifiasm ( Cheng *et al.*, 2021); haplotypic duplication was identified and removed with purge_dups ( Guan *et al.*, 2020). One round of polishing was performed by aligning 10X Genomics read data to the assembly with longranger align, calling variants with freebayes ( Garrison & Marth, 2012). The assembly was then scaffolded with Hi-C data ( Rao *et al.*, 2014) using SALSA2 ( Ghurye *et al.*, 2019). The assembly was checked for contamination and corrected using the gEVAL system ( Chow *et al.*, 2016) as described previously ( Howe *et al.*, 2021). Manual curation ( Howe *et al.*, 2021) was performed using gEVAL, HiGlass ( Kerpedjiev *et al.*, 2018) and Pretext. The mitochondrial genome was assembled using MitoHiFi ( Uliano-Silva *et al.*, 2021). The genome was analysed and BUSCO scores generated within the BlobToolKit environment ( Challis *et al.*, 2020). Table 3 contains a list of all software tool versions used, where appropriate.

**Table 3.** Software tools used.

Software tool	Version	Source
Hifiasm	0.12	( Cheng *et al.*, 2021)
purge_dups	1.2.3	Guan *et al.*, 2020
SALSA2	2.2	Ghurye *et al.*, 2019
longranger align	2.2.2	https://support.10xgenomics.com/genome-exome/ software/pipelines/latest/advanced/other-pipelines
freebayes	1.3.1-17-gaa2ace8	Garrison & Marth, 2012
MitoHiFi	1.0	( Uliano-Silva *et al.*, 2021)
gEVAL	N/A	Chow *et al.*, 2016
HiGlass	1.11.6	( Kerpedjiev *et al.*, 2018)
PretextView	0.1.x	https://github.com/wtsi-hpag/PretextView
BlobToolKit	2.6.2	Challis *et al.*, 2020

### Ethics/compliance issues

The materials that have contributed to this genome note have been supplied by a Darwin Tree of Life Partner. The submission of materials by a Darwin Tree of Life Partner is subject to the Darwin Tree of Life Project Sampling Code of Practice. By agreeing with and signing up to the Sampling Code of Practice, the Darwin Tree of Life Partner agrees they will meet the legal and ethical requirements and standards set out within this document in respect of all samples acquired for, and supplied to, the Darwin Tree of Life Project. Each transfer of samples is further undertaken according to a Research Collaboration Agreement or Material Transfer Agreement entered into by the Darwin Tree of Life Partner, Genome Research Limited (operating as the Wellcome Sanger Institute), and in some circumstances other Darwin Tree of Life collaborators.

## Data availability

European Nucleotide Archive: Taurulus bubalis (long-spined sea scorpion). Accession number PRJEB45118; https://identifiers.org/ena.embl/PRJEB45118.

The genome sequence is released openly for reuse. The *T. bubalis* genome sequencing initiative is part of the Darwin Tree of Life (DToL) project. All raw sequence data and the assembly have been deposited in INSDC databases. The genome will be annotated using the RNA-Seq data and presented through the Ensembl pipeline at the European Bioinformatics Institute. Raw data and assembly accession identifiers are reported in Table 1.
