# Supplementary material for: The genome sequence of the long-spined sea scorpion, Taurulus bubalis (Euphrasén, 1786)
Source: Wellcome Open Res. 2021 Nov 8;6:299. [Version 1] doi: 10.12688/wellcomeopenres.17356.1 (PMC9587380; doi:10.12688/wellcomeopenres.17356.1)
Supplement: Peer review history

# Peer review: Reviewer response for version 1

**DOI:** 10.21956/wellcomeopenres.19189.r52948

**Published:** 2022-10-21

**Authors:** Thomas Desvignes

**Affiliations:** 1 University of Oregon, Eugene, USA

In this article, the authors report on the publicly-available, chromosome-scale genome assembly of the longspined bullhead *Taurulus bubalis*, a marine sculpin from western Europe. The assembly appears to be of great quality using appropriate methods and following the high standard of the Darwin Tree of Life Project. I have only minor comments.

 In the abstract the sequenced specimen is referred to as a female, but the first sentence of the genome sequence report and of the methods state that the specimen was of unknown sex. What is the accurate situation? Knowing the sex of the individual would obviously be beneficial.

 Is there any information on the karyotype of *T. bubalis* or closely related species to support that the 21 pseudochromosome molecules obtained is the expected number of chromosomes?

 I am confused by the statement that the 21 chromosomal-level scaffolds represent “21 autosomes”? What about potential sex chromosomes? Is there any information supporting the hypothesis that the species’ sex determination mechanism is environmental? Or is it instead that the analysis of the two haplotypes did not reveal obvious differences in any chromosome pairs? In which case, if the species has a genetically determined sex, the potential sex chromosomes would be homomorphic, although still not autosomes, I believe.

 According to Fishbase, “longspined bullhead” is the FAO-accepted common name for *Taurulus bubalis* while “longspined sea-scorpion” is a vernacular name from Ireland and the UK. Please verify and if appropriate replace “sea-scorpion” by “bullhead”, also note that there is apparently no hyphen in “longspined”.

 Similarly, based on Fishbase, the FAO-accepted common name for *Myoxocephalus scorpius* is “shorthorn sculpin” while “bull-rout” is considered a vernacular name.

 In the background section, the mentioned spines are preopercular spines. It might be worth simply adding this word because these preopercular spines represent a distinctive character in sculpins.

Are sufficient details of methods and materials provided to allow replication by others?

Yes

Is the rationale for creating the dataset(s) clearly described?

Yes

Are the datasets clearly presented in a useable and accessible format?

Yes

Are the protocols appropriate and is the work technically sound?

Yes

Reviewer Expertise:

Fish evolutionary biology.

I confirm that I have read this submission and believe that I have an appropriate level of expertise to confirm that it is of an acceptable scientific standard.

# Peer review: Reviewer response for version 1

**DOI:** 10.21956/wellcomeopenres.19189.r52365

**Published:** 2022-09-20

**Authors:** Bing-Jian Liu

**Affiliations:** 1 National Engineering Laboratory of Marine Germplasm Resources Exploration and Utilization, Zhejiang Ocean University, Zhejiang, China

In this study, the authors have generated a chromosome-scale genome assembly of the long-spined sea scorpion, and the protocols for the genome assembly are well chosen and robust. The Hi-C contact heat map and BUSCO results are quite good, showing a high-quality reference genome.

 I notice that the gender of the sample was unknown. Why not determine the gender of the sample? In my opinion, gender is a key piece of information for a genome. Of course, I know very little about the biological characteristics of *Taurulus bubalis*. So if the comment is not appropriate, please ignore it.

Are sufficient details of methods and materials provided to allow replication by others?

Yes

Is the rationale for creating the dataset(s) clearly described?

Yes

Are the datasets clearly presented in a useable and accessible format?

Yes

Are the protocols appropriate and is the work technically sound?

Yes

Reviewer Expertise:

population genetics/genomics

I confirm that I have read this submission and believe that I have an appropriate level of expertise to confirm that it is of an acceptable scientific standard.
